# Identifying Risk Factors Associated with Major Complications and Refractory Course in Patients with Osteomyelitis of the Jaw: A Retrospective Study

**DOI:** 10.3390/jcm12144715

**Published:** 2023-07-17

**Authors:** Mathilde Fenelon, Steven Gernandt, Romain Aymon, Paolo Scolozzi

**Affiliations:** 1 Division of Oral and Maxillofacial Surgery, Department of Surgery, Geneva University Hospitals, 1205 Geneva, Switzerland; mathilde.fenelon@hcuge.ch (M.F.); steven.gernandt@hcuge.ch (S.G.); romain.aymon@hcuge.ch (R.A.); 2 UFR des Sciences Odontologiques, University Bordeaux, 33000 Bordeaux, France; 3 Service de Chirurgie Orale, CHU de Bordeaux, 33000 Bordeaux, France

**Keywords:** bone, osteomyelitis, decortication, treatment outcome, oral and maxillofacial surgery

## Abstract

Despite improved knowledge regarding the diagnosis and treatment of osteomyelitis of the jaw (OMJ), it remains a clinical challenge for oral and maxillofacial surgeons. This study aimed to identify risk factors associated with severe forms of OMJ, i.e., related to the occurrence of major complications or the refractory course of the disease. A retrospective study was performed based on the medical records of all patients diagnosed with OMJ from the past 20 years. Collected data included demographic information, medical and dental history, clinical, radiological, and bacterial findings as well as treatment modalities. The main outcome variables were the onset of major complications and treatment results. Fifty-four patients were included. Our results showed that alcohol and smoking habits, as well as malnutrition, were significantly associated with the occurrence of major complications. We also established that dental implant-induced OMJ should be considered an aggressive subtype of OMJ. Finally, clinical bone exposure was significantly associated with unfavorable outcomes, whereas dental causes or radiological evidence of periosteal reaction were predictive of successful outcomes. Identifying such factors could be useful in preventing serious complications and informing patients about the refractory course of the disease based on the presence of these factors.

## 1. Introduction

Osteomyelitis is defined as a progressive inflammatory process involving the bone and bone marrow. This inflammatory condition of the bone begins in the medullar cavity and, with progression, extends to involve the Haversian systems, the periosteum, and eventually the overlying soft tissue of the affected area [1,2]. The mandible is the most frequently affected bone within the facial skeleton [2,3]. Despite the advent of antibiotics and the improvement of dental care, osteomyelitis of the jaw (OMJ) is still encountered in developed countries [4].

OMJ is a complex entity with various clinical or radiological features, etiology, and pathogenesis, resulting in several terminologies and classification systems [5,6,7,8]. The most commonly used is the Zurich classification system, which divides the disease into three distinct entities: acute osteomyelitis, secondary chronic osteomyelitis (SCO), and primary chronic osteomyelitis (PCO) [2,7]. This classification is based on the clinical appearance and course of the disease as well as radiological features. Acute osteomyelitis differs from SCO by the arbitrary time limit of 4 weeks after onset of the disease. Thus, acute osteomyelitis and SCO are usually considered the same disease at different stages of their course [2,4]. They are characterized by suppuration, fistula and/or bony sequestrum, and represent a true bacterial infection of the jawbone, whereas PCO is a nonsuppurative inflammation of unknown etiology, without fistula formation or bony sequestra [1,7].

The treatment of acute and chronic osteomyelitis usually involves a surgical approach combined with long-term broad-spectrum antibiotic therapy, as well as nonsteroidal anti-inflammatory drugs to control symptoms [4,8,9,10,11]. The surgical debridement of infected tissue depends on the extent of the lesion and varies from conservative treatment (i.e., removal of affected teeth or implants, sequestrectomy, local curettage or decortication) to more aggressive surgical procedures (i.e., marginal or segmental resection) [2,11,12,13]. Standard antibiotic protocols to treat osteomyelitis usually involve 6 weeks of post-operative intravenous and/or oral administration [8,14].

Despite improved knowledge regarding the diagnosis and treatment of osteomyelitis, its clinical course often involves protracted treatment and increased surgical morbidity, thus remaining a clinical challenge for oral and maxillofacial surgeons [3,15]. Furthermore, the clinician has to also deal with major complications that occur in patients with OMJ such as life-threatening deep neck abscesses, cutaneous fistulae, or pathological fractures of the weakened bone by infection [1,16,17].

Some authors have discussed the existence of predisposing factors associated with OMJ development. Local factors, mainly dental infection or trauma, and systemic factors, such as immunodeficiencies or diabetes, have thus been frequently associated with the onset of OMJ [1,9,18,19,20,21].

However, few studies have focused on the association between risk factors and the occurrence of complications or the refractory course of OMJ [1,22]. The aim of the present study was thus to identify possible general and local risk factors associated with the occurrence of major complications and/or a refractory course in patients diagnosed with OMJ. In order to achieve the proposed objective, we hypothesized that some medical conditions as well as local clinical, radiological, or bacteriological features could be associated with an increased risk of severe OMJ. Identifying such factors might help the clinician to better prevent and manage this insidious disease.

## 2. Materials and Methods

### 2.1. Study Design and Sample

To address this research purpose, the investigators designed and implemented a retrospective cohort study. The present study followed the Declaration of Helsinki guidelines and was approved by the ethics commission of the University Hospitals of Geneva (approval no. 2022-01756). The Strengthening the Reporting of Observational Studies in Epidemiology (STROBE) Checklist was used to design this observational study and to report results.

The study population was selected from a database of patients diagnosed with OMJ and treated in the division of Oral and Maxillofacial Surgery at the University Hospitals of Geneva, Switzerland, over the past twenty years.

All patients with a diagnosis of OMJ confirmed by histological analysis and with complete medical records were included in this study without age limitation. Patients were excluded if they had a history of head and neck irradiation, antiresorptive medication usage, and/or anti-angiogenic agents. Patients with a history of malignant pathology of the oral cavity or malignant disease with metastasis to the jaws were also excluded.

### 2.2. Variables

The main outcome variables were the onset of major complications and treatment outcomes (i.e., successful treatment versus no or partial resolution). Major complications involved pathological fractures, deep neck abscesses, and cutaneous fistulae. Successful treatment was defined as an absence of persistent clinical symptoms and no radiographic evidence of bony disease progression [1,14].

Other variables included demographic data, medical and dental history, smoking habits, initial clinical presentation, symptoms duration (time from start of symptoms until first visit to our institute), type of osteomyelitis (according to the Zurich classification), microbiological information (including bacterial species and information regarding the bacterial sample collection method: intraorally or from an extraoral approach), radiological findings, treatment modalities (including procedure, number of surgeries, antibiotic treatment and outcomes), and follow-up duration.

### 2.3. Data Analysis

All statistical analysis was performed using R statistical software (version 4.2.1; R Development Core Team, Vienna, Austria). Patient characteristics were summarized using descriptive statistics, with mean and standard deviation reported for normally distributed continuous variables, median and interquartile range (IQR) reported for non-normally distributed variables and counts and percentages reported for categorical variables. Due to small cell counts, we used Fisher’s exact test to analyze bivariate associations between categorical variables and the Kruskal–Wallis test for non-normally distributed continuous variables. Significance level was set at *p* ≤ 0.05.

## 3. Results

Fifty-four patients diagnosed with OMJ from 2002 to 2022 were included in this study (Table 1).

Among the 54 cases of OMJ, seven were acute osteomyelitis (13%), 35 were SCO (64.8%), and 12 were PCO (22.2%), one of which was a case of SAPHO syndrome (synovitis, acne, pustulosis, hyperostosis, osteitis).

Twenty-nine patients were female (53.7%) and the average age at onset of the disease was 47.5 years old (±18.9). Seven patients (13%) had a history of diabetes, and 18 patients (33.3%) had a history of cardiovascular disease including hypertension. Five patients (9.3%) were immunocompromised, four patients (7.4%) had been previously diagnosed with inflammatory rheumatic diseases, and three patients (5.6%) suffered from malnutrition. An allergy to amoxicillin was reported in nine patients (16.7%). Finally, 27 patients (50%) were smokers, eleven patients (20.4%) had alcohol habits, and four patients (7.4%) reported illicit drug use.

Symptoms duration ranged from 1 week to 7 years with a median of 2 months (IQR: 1–9 months). Most of the included patients (44 patients, 81.5%) reported recent oral or dental treatment. Seven patients suffered from dental infections (15.9%), tooth extraction was performed in 23 patients (52.3%), dental care in four patients (9.1%), nine patients (20.5%) had recent dental implant placement and six patients had a mandibular trauma (13.6%). None of the included patients reported previous jaw osteotomies. Unknown or unclear etiology was found in ten patients.

The most common initial clinical findings were pain and swelling (77.8 and 66.7% of patients, respectively). Trismus was reported in 19 patients (35.5%), suppuration in 18 patients (33.3%), and neurosensory change in 11 patients (20.4%). Buccal or cutaneous fistula was observed in seven patients (13%) and three patients had exposed bone (5.6%).

**Figure 1:**
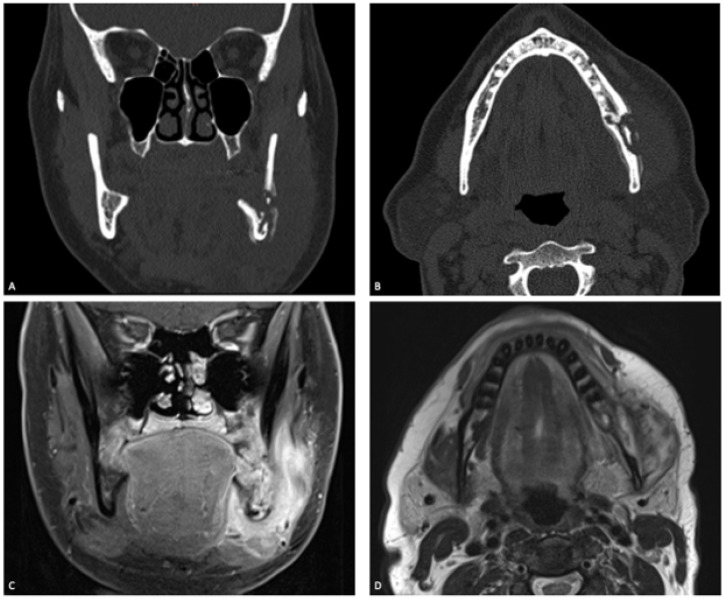
Coronal (**A**) and axial (**B**) CT-scans of a 52-year-old woman with secondary chronic osteomyelitis of the mandible. Osteolysis, sequestration, and periosteal reaction were shown on the left side of the mandible. Coronal (**C**) and axial (**D**) MRIs showed the soft tissue involvement with signs of myositis of the left masseter and mylohyoid muscles.

Diagnostic imaging of the patients was carried out with one or more radiological examinations: computed tomography (CT) scans (47, 87.0%), magnetic resonance imaging (MRI) (33, 61.1%), panoramic radiographs (21, 38.9%), CBCT (6, 11.1%), scintigraphy (4, 7.4%) and positron emission tomography-CT (PET-CT) (4, 7.4%) (Figure 1). Osteolysis was the most common radiographic finding (48, 88.9%), followed by osteosclerosis (32, 59.3%), periosteal reaction (32, 59.3%), and muscle infiltration (27, 50.0%) (Figure 1).

Cortical destruction was observed in 27 patients (50.0%), bone sequestra in 23 (42.6%) and delayed bone healing in 12 patients (22.2%). Bone hypertrophy and bone enhancement (scintigraphy) were also detected in a few patients (*n* = 3, 5.6% and *n* = 4, 7.4% respectively). Finally, pathologic fractures were observed in seven patients (13.0%). OMJ affected the mandible in 51 patients and the location of mandibular OMJ was distributed as follows: body (40, 74.1%), angle (23, 42.6%), ramus (11, 20.4%), symphysis (11, 20.4%), condyle (*n* = 3, 5.6%), coronoid process (2, 3.7%).

Bacteriological cultures were carried out using an intraoral approach in 29 patients (54.7%) and a cervical approach in 24 patients (45.3%). The streptococcus (S.) milleri group, which consists of three species, *S. constellatus*, *S. intermedius*, and *S. anginosus*, was frequently isolated (22, 40.7%), followed by the S. mitis group (14, 26.2%). Other isolated germs were: Actinomyces (8, 15.1%), Staphylococcus epidermidis (4, 7.5%), Fusobacterium (2, 3.8%), Prevotella (2, 3.8%), Veillonella (1, 1.9%), Parvimonas micra (1, 1.9%), Eikenella corrodens (2, 3.8%), Klebsiella oxytoca (1, 1.9%), Campylobacter rectus (1, 1.9%), Staphylococcus capitis (2, 3.8%), Escherichia coli (1, 1.9%), and Micrococcus luteus (1, 1.9%). In the PCO group, nine patients (75%) had sterile bacteriological samples.

OMJ were complicated by a deep neck abscess, pathologic fracture, or cutaneous fistula in 17 patients (31.5%). Among them, 12 patients had SCO, four patients had acute OMJ, and only one patient had PCO. Alcohol and smoking habits (*p* = 0.025 and 0.018, respectively), as well as malnutrition (*p* = 0.027), were significantly associated with the occurrence of complications (Table 2). Implant-associated OMJ was another factor significantly associated with such complications (*p* = 0.003). Finally, they also showed a significant association with short symptoms duration (*p* = 0.016).

Twenty-nine patients (53.7%) received previous treatment before referral: antibiotics (25, 86.2%), non-steroidal anti-inflammatory drugs (NSAID) and/or steroids (6, 20.7%), surgery (10, 34.5%), and hyperbaric oxygen therapy (1, 3.4%). Once OMJ was diagnosed in our department, almost all patients received both medical and surgical treatment (49, 90.7%). An antibiotic therapy was administered in 53 patients (98.1%) with a median duration of two months (IQR: 1.5–3.6) (Table 3).

The most commonly administered antibiotics were amoxicillin plus clavulanic acid (38, 71.7%) and clindamycin (24, 45.3%). Four patients did not require surgical treatment. Twenty-seven patients underwent a single surgery (54%). Decortication was the first line of treatment in 30 patients (60.0%). First surgeries also included curettage (17, 34.0%), tooth extraction (17, 34.0%), osteosynthesis (12, 24.0%), incision and abscess drainage (12, 24.0%), sequestrectomy (10, 20.0%), removal of hardware or associated implants (10, 20.0%), and debridement (2, 4.0%). Initial segmental resection (i.e., interruptive) was necessary in three patients (6.0%), in combination with an iliac bone graft as the reconstruction method in one patient (2.0%).

Sixteen patients had two surgeries (32%) and seven patients had three or more surgeries (14%). Additional surgeries consisted of decortication (10, 43.5%), curettage (9, 39.1%), removal of hardware or associated implants (9, 39.1%), tooth extraction (7, 30.4%), incision and abscess drainage (7, 30.4%), osteosynthesis (6, 26.1%), sequestrectomy (4, 17.4%), partial or segmental resection (2, 8.7% and 3, 13.0%, respectively) associated with iliac bone graft in three patients (13.0%).

Follow-up duration ranged from one month to eleven years with a median of 1.5 years (IQR: 1–3 years). Successful outcomes were obtained in 48 patients (88.9%), whereas partial or no improvements were observed in six patients. Some factors were significantly associated with successful treatment outcome such as a recent dental history (meaning oral pain, care or surgery) and radiological evidence of a periosteal reaction (*p* ≤ 0.05). On the contrary, clinical bone exposure was significantly associated with unfavorable outcomes (*p* ≤ 0.05). A trend towards significance was observed for patients with an amoxicillin allergy that was frequently associated with unsuccessful treatment (*p* = 0.051). Finally, other possible risk factors, such as longer symptom duration (8.0 months [5.2, 11.5]) and radiological evidence of osteosclerosis, were frequently observed in patients with unsuccessful treatment, without reaching statistical significance (*p* = 0.063 and *p* = 0.071, respectively) (Table 4).

## 4. Discussion

This retrospective study described and analyzed the data from a series of patients with OMJ. The specific aim of this study was to identify risk factors statistically associated with severe forms of OMJ.

The author’s hypothesis was that certain medical conditions or local clinical, radiological, or bacteriological features could be associated with an increased risk of major complications or refractory course of OMJ. The results of the present study have substantially confirmed our hypothesis. First, the results emphasized the existence of general (alcohol and smoking habits, as well as malnutrition) and local factors (dental implant–induced OMJ) associated with the occurrence of major complications (i.e., characterized by deep neck space abscess formation, extraoral cutaneous fistula, and pathological fractures). Secondly, bone exposure was the only risk factor associated with the refractory course of the disease, whereas dental etiology and radiological evidence of a periosteal reaction were associated with successful treatment. Moreover, a trend towards significance was observed for patients with an amoxicillin allergy, longer symptom duration, and radiological evidence of osteosclerosis that were frequently associated with unsuccessful treatment. Finally, regarding microbiological results, there was no association between isolated species from bacteriological samples and OMJ severity. To the best of our knowledge, this is the largest retrospective study investigating both general and local factors associated with severe forms of OMJ.

In the present study, one-third of the patients developed major complications related to OMJ such as deep neck abscess, pathologic fractures, or cutaneous fistula. Our results showed that alcohol and smoking habits, as well as malnutrition, were significantly associated with the occurrence of major complications. To our knowledge, this is the first study investigating risk factors associated with such complications. These factors (alcohol and smoking habits, as well as malnutrition) have been previously identified only as predisposing factors to develop OMJ [9,17,19]. Other predisposing systemic conditions for the onset of the disease have been reported, such as diabetes mellitus, anemia, and intravenous drug abuse [9]. In the present study, they were not associated with a higher risk of complication.

Another interesting risk factor identified in this study is implant-associated OMJ. Among the 17 patients who developed complications, 41.2% of them were related to recent dental implant placement. Dental implant–induced OMJ is a rare disease, with an estimated incidence of 0.02% [23], that has not been reported extensively. Available studies on dental implant-induced OMJ described it as an aggressive subtype of OMJ with short symptom duration and rapid evolution toward serious complications such as a pathologic fracture and deep neck abscess [17,24,25,26]. Yahalom et al. also reported that previous dental implants were predictive factors for OMJ progression, primary treatment failure, and the need for further aggressive surgical procedures compared to other OMJ etiologies [23]. Aggressiveness of dental implant–induced OMJ could be explained by a bacterial infection induced during dental implant insertion. Furthermore, the dental implant’s surface might favor bacterial adhesion and accumulation, causing a rapidly increasing inflammatory response [27,28].

Osteomyelitis is known to be a challenging disease to treat. Success rates vary widely across studies and complete remission rate ranges from 22.5 to 91.7% [4,13]. In the present study, successful outcomes were obtained in 48 patients (88.9%). Almost all patients received both medical (antibiotic therapy and/or NSAIDs) and surgical treatment. Decortication was the procedure of choice. Decortication was not performed in the first place mainly when the patient first required surgical treatment for an infectious complication or when the lesion was limited, thereby allowing only a local curettage or, on the contrary, when the lesion was very extensive and required a segmental resection. In the present study, 54% of patients underwent a single surgery and 38% had two to three interventions. Few studies mentioned the average number of surgeries per patient. In their retrospective study of 30 patients, Baltensperger et al. performed zero to six surgical procedures (mean: 2.2), with decortication being the procedure of choice in 80% of cases. Complete remission was observed in 36.7% of patients [7]. Similarly, a retrospective study of 10 patients with PCO and 12 patients with SCO reported an average number of surgical procedures per patient of 2.9 and 2.75, respectively [4]. Decortication was performed in 60% of patients. Symptom relief was achieved in 50% of patients with PCO and 91.7% of patients with SCO. Jia et al. also performed a decortication in the first place in 94.5% of patients and 33.8% of them underwent a second decortication. Symptom relief was achieved in 54.4% of patients [29].

Despite relatively standard surgical approaches and a wide variety of antibiotic choices, the clinical course of OMJ often leads to unfavorable outcomes. We thus hypothesized that either certain conditions or clinical, radiological, and bacteriological characteristics could lead to unfavorable outcomes. First, we observed that clinical bone exposure was significantly associated with a refractory course of the disease. This could be explained by the persistent exposure of bone to microorganisms from the oral cavity, thereby making it more difficult to completely eradicate these pathogens. Bone exposure is commonly observed in patients with jaw osteonecrosis caused by head and neck radiotherapy or antiresorptive medication [30]. However, this clinical feature is very rarely reported in studies focusing on OMJ [9], thus preventing us from comparing our results with those of the literature. Our results also showed that radiological evidence of a periosteal reaction was significantly associated with successful treatment, whereas radiological evidence of osteosclerosis was frequently observed in patients with unsuccessful outcomes without reaching significance. Both primary and secondary osteomyelitis may demonstrate these radiological patterns (periosteal reaction and osteosclerosis) on diagnostic imaging [2,4]. Periosteal reaction is a nonspecific reactive inflammatory phenomenon leading to the formation of new bone outside the normal cortical layer. This subperiosteal bone formation is more frequently observed in younger individuals and in an early stage of OMJ, whereas osteosclerosis are more prominent in elderly patients and in advanced stages of the disease [2]. Moreover, a trend towards significance was observed for patients with an amoxicillin allergy and longer symptom duration that were frequently associated with unsuccessful treatment. Finally, our study highlighted that a recent dental history was significantly associated with successful treatment. Identification of an underlying dental cause of OMJ may lead to better outcomes by addressing the odontogenic foci more readily. Indeed, conservation of pathogenic teeth, usually caused by the delayed recognition of the infection foci, is considered to be a risk factor for OMJ recurrence [22].

The main strength of the present study was that ours is one of the largest series investigating risk factors associated with severe forms of OMJ. To our knowledge, only two studies previously investigated possible variables associated with treatment outcome of OMJ [1,22]. One study investigated risk factors for recurrence in patients with SCO [22]. Factors such as age, pre-admission antibiotic administration, and conservation of pathogenic teeth were found to be risk factors for recurrence. Unfortunately, this study did not consider local clinical and radiological features of OMJ, thereby preventing us from comparing our results to this study. Finally, only one study investigated similar parameters to our study (i.e., medical conditions, clinical, radiological, and bacteriological features). Haeffs et al. only reported that psychiatric disorders were statistically associated with unsuccessful initial treatment [1]. This result was not observed in our study. This may be related to the larger proportion of their sample presenting a psychiatric disorder compared to our study (45.2% versus 16.7%, respectively).

Some limitations of this study must be taken into consideration. This is a retrospective study with a limited number of patients. Although OMJ usually shows specific clinical and radiological patterns, we decided to only include patients whose OMJ was confirmed by histopathology. Histological examination of biopsies remains necessary to confirm the diagnosis in cases of unclear clinical and radiological features, and furthermore to exclude possible differential diagnoses [2]. With a larger sample size, the relationship between variables, such as unsuccessful treatment and amoxicillin allergy, longer symptom duration, or osteosclerosis, could have reached levels of statistical significance.

## 5. Conclusions

This study described the clinical, radiological, and bacteriological findings, treatment, and outcomes in 54 patients with osteomyelitis of the jaws treated during the past 20 years in the University Hospitals of Geneva. This enabled the identification of risk factors statistically associated with severe forms of OMJ. Our results showed that alcohol and smoking habits, as well as malnutrition, were significantly associated with the occurrence of major complications. We also established that dental implant-induced OMJ should be considered an aggressive subtype of OMJ that can evolve rapidly towards serious complications. Finally, we observed that bone exposure was significantly associated with unfavorable outcomes, whereas dental etiology and radiological evidence of periosteal reaction were predictive of successful treatment. Identifying such factors could be useful for a better management of the patient, by identifying patients at high risk of major complications or who might be refractory to conventional treatments and thus initiate thorough monitoring. However, further studies with larger sample sizes are needed to reinforce these preliminary results.

## Figures and Tables

**Table 1:** General characteristics of included patients with osteomyelitis.

General Characteristics of the Population		*n* (%)
Age in years (mean (SD))		47.5 (18.9)
Sex = H		25 (46.3)
Osteomyelitis	Acute osteomyelitis	7 (13.0)
	Secondary chronic osteomyelitis (SCO)	35 (64.8)
	Primary chronic osteomyelitis (PCO)	12 (22.2)
Medical condition	Diabetes	7 (13.0)
	Hypercholesterolemia	5 (9.3)
	Cardiovascular disease (Incl. hypertension)	18 (33.3)
	Psychiatric disorder	9 (16.7)
	Anemia	2 (3.7)
	Malnutrition	3 (5.6)
	Bone disorder	4 (7.4)
	Immunosuppressed state	5 (9.3)
	Inflammatory rheumatic diseases	4 (7.4)
	Allergy to amoxicillin	9 (16.7)
	Illicit drug use	4 (7.4)
	Alcohol habit	11 (20.4)
	Smoking habit	27 (50.0)
Disease history	Recent oral care or surgery or tooth infection	37 (68.5)
	Recent implant placement	9 (16.7)
	Symptoms duration in months (median [IQR])	2.0 [1.0, 9.0]
Initial clinical presentation	Fever	3 (5.6)
	Pain	42 (77.8)
	Swelling	36 (66.7)
	Suppuration	18 (33.3)
	Neurosensory change	11 (20.4)
	Trismus	19 (35.2)
	Fistula	7 (13.0)
	Bone exposure	3 (5.6)
	Deep neck abscess	9 (16.7)
Radiographic findings	Osteolysis	48 (88.9)
	Bony sequestrum	23 (42.6)
	Periosteal reaction	32 (59.3)
	Cortical destruction	27 (50.0)
	Osteosclerosis	32 (59.3)
	Delayed bone healing	12 (22.2)
	Collection	12 (22.2)
	Pathologic fracture	7 (13.0)
	Myositis/Muscle infiltration	27 (50.0)
	Bone hypertrophy	3 (5.6)

IQR: interquartile range; SD: Standard deviation.

**Table 2:** Data related to the occurrence of complications in patients with osteomyelitis.

Variable		Complication		*p* Value
		No (*n* = 37)	Yes (*n* = 17)	
Age in years (mean (SD))		45.0 (20.3)	53.0 (14.3)	0.150
Sex = H		17 (45.9)	8 (47.1)	1.000
Osteomyelitis				0.068
classification	Acute osteomyelitis	3 (8.1)	4 (23.5)	-
	Secondary chronic osteomyelitis	23 (62.2)	12 (70.6)	-
	Primary chronic osteomyelitis	11 (29.7)	1 (5.9)	-
Medical condition	Diabetes	3 (8.1)	4 (23.5)	0.189
	Hypercholesterolemia	3 (8.1)	2 (11.8)	0.645
	Cardiovascular disease (Incl. hypertension)	11 (29.7)	7 (41.2)	0.536
	Psychiatric disorder	6 (16.2)	3 (17.6)	1.000
	Anemia	1 (2.7)	1 (5.9)	0.535
	Malnutrition	0 (0.0)	3 (17.6)	0.027
	Bone disorder	2 (5.4)	2 (11.8)	0.582
	Immunosuppressed state	2 (5.4)	3 (17.6)	0.311
	Inflammatory rheumatic diseases	4 (10.8)	0 (0.0)	0.296
	Allergy to amoxicillin	5 (13.5)	4 (23.5)	0.439
	Illicit drug use	3 (8.1)	1 (5.9)	1.000
	Alcohol habit	4 (10.8)	7 (41.2)	0.025
	Smoking habit	14 (37.8)	13 (76.5)	0.018
Disease history	Recent oral care or surgery or tooth infection	28 (75.7)	9 (52.9)	0.121
	Recent implant placement	2 (5.4)	7 (41.2)	0.003
	Symptoms duration in months (median [IQR])	2.0 [1.0, 12.0]	1.0 [1.0, 2.0]	0.016
Initial clinical	Fever	3 (8.1)	0 (0.0)	0.544
presentation	Pain	29 (78.4)	13 (76.5)	1.000
	Swelling	24 (64.9)	12 (70.6)	0.763
	Suppuration	10 (27.0)	8 (47.1)	0.215
	Neurosensory change	5 (13.5)	6 (35.3)	0.081
	Trismus	10 (27.0)	9 (52.9)	0.076
	Fistula	3 (8.1)	4 (23.5)	0.189
	Bone exposure	2 (5.4)	1 (5.9)	1.000
Radiographic	Osteolysis	33 (89.2)	15 (88.2)	1.000
findings	Bony sequestrum	15 (40.5)	8 (47.1)	0.769
	Periosteal reaction	22 (59.5)	10 (58.8)	1.000
	Cortical destruction	19 (51.4)	8 (47.1)	1.000
	Osteosclerosis	24 (64.9)	8 (47.1)	0.246
	Delayed bone healing	9 (24.3)	3 (17.6)	0.732
	Collection	6 (16.2)	6 (35.3)	0.162
	Myositis/Muscle infiltration	16 (43.2)	11 (64.7)	0.241
	Bone hypertrophy	3 (8.1)	0 (0.0)	0.544

**Table 3:** Treatment modalities and outcomes of patients with osteomyelitis.

Treatment Variables		Data
Surgery	Decortication (*n* (%))	40 (74.1%)
	Number of surgery	
	0	4
	1	27
	2	16
	3 or more	7
Antibiotic	Number of patient (*n* (%))	53 (98.1)
	Treatment duration in month (median [IQR])	2.0 [1.5, 3.6]
NSAID treatment	Yes (*n* (%))	35 (64.8)
Treatment outcomes	Successful treatment (*n* (%))	48 (88.9)
	Partial or unsuccessful treatment	6 (11.1)
Follow-up duration (in years)		1.5 [1.0, 3.0]

**Table 4:** Data related to the treatment outcomes in patients with osteomyelitis.

Variable		Partial Response or Unsuccessful Treatment	Successful Treatment	*p* Value
		(*n* = 6)	(*n* = 48)	
Age in years (mean (SD))		50.3 (22.0)	47.1 (18.7)	0.706
Sex = H		2 (33.3)	23 (47.9)	0.675
Osteomyelitis				0.679
classification	Acute osteomyelitis	0 (0.0)	7 (14.6)	-
	Secondary chronic osteomyelitis	4 (66.7)	31 (64.6)	-
	Primary chronic osteomyelitis	2 (33.3)	10 (20.8)	-
Medical condition	Diabetes	1 (16.7)	6 (12.5)	1.000
	Hypercholesterolemia	1 (16.7)	4 (8.3)	0.459
	Cardiovascular disease (Incl. hypertension)	2 (33.3)	16 (33.3)	1.000
	Psychiatric disorder	2 (33.3)	7 (14.6)	0.259
	Anemia	0 (0.0)	2 (4.2)	1.000
	Malnutrition	0 (0.0)	3 (6.2)	1.000
	Bone disorder	0 (0.0)	4 (8.3)	1.000
	Immunosuppressed state	0 (0.0)	5 (10.4)	0.934
	Inflammatory rheumatic diseases	1 (16.7)	3 (6.2)	0.385
	Allergy to amoxicillin	3 (50.0)	6 (12.5)	0.051
	Illicit drug use	0 (0.0)	4 (8.3)	1.000
	Alcohol habit	1 (16.7)	10 (20.8)	0.385
	Smoking habit	2 (33.3)	25 (52.1)	0.669
Disease history	Recent history of oral pain, oral care or surgery			0.015
	Yes	3 (50.0)	41 (85.4)	-
	No	3 (50.0)	4 (8.3)	-
	Unclear	0 (0.0)	3 (6.2)	-
	Recent implant placement	0 (0.0)	9 (18.8)	0.574
	Symptoms duration in months (median [IQR])	8.0 [5.2, 11.5]	2.0 [1.0, 6.0]	0.063
Initial clinical	Fever	0 (0.0)	3 (6.2)	1.000
presentation	Pain	5 (83.3)	37 (77.1)	1.000
	Swelling	3 (50.0)	33 (68.8)	0.388
	Suppuration	2 (33.3)	16 (33.3)	1.000
	Neurosensory change	1 (16.7)	10 (20.8)	1.000
	Trismus	2 (33.3)	17 (35.4)	1.000
	Fistula	1 (16.7)	6 (12.5)	1.000
	Bone exposure	2 (33.3)	1 (2.1)	0.030
Radiographic	Osteolysis	5 (83.3)	43 (89.6)	0.525
findings	Bony sequestrum	1 (16.7)	22 (45.8)	0.224
	Periosteal reaction	1 (16.7)	31 (64.6)	0.036
	Cortical destruction	4 (66.7)	23 (47.9)	0.669
	Osteosclerosis	6 (100.0)	26 (54.2)	0.071
	Delayed bone healing	1 (16.7)	11 (22.9)	1.000
	Collection	1 (16.7)	11 (22.9)	1.000
	Myositis/Muscle infiltration	4 (66.7)	23 (47.9)	0.669
	Bone hypertrophy	1 (16.7)	2 (4.2)	0.303

IQR: interquartile range; SD: Standard deviation.

## Data Availability

The authors declare that the data supporting the findings of this study are available within the paper.
